# Altered Expression of EMT-Related Factors Snail, Wnt4, and Notch2 in the Short-Term Streptozotocin-Induced Diabetic Rat Kidneys

**DOI:** 10.3390/life12101486

**Published:** 2022-09-25

**Authors:** Matea Dragun Jurić, Anita Racetin, Natalija Filipović, Nela Kelam, Sandra Kostić, Dragan Ljutić, Katarina Vukojević

**Affiliations:** 1 Intensive Care Unit of the Department of Internal Medicine, University Hospital in Split, 21000 Split, Croatia; 2 Department of Anatomy, Histology and Embryology, University of Split School of Medicine, 21000 Split, Croatia; 3 Department of Nephrology, University Hospital in Split, 21000 Split, Croatia

**Keywords:** diabetic nephropathy, epithelial to mesenchymal transition (EMT), snail, Notch2, Wnt4

## Abstract

Background: The aim of this study was to determine the expression of epithelial to mesenchymal transition (EMT)-related transcription factors Snail, Wnt4, and Notch2 with key roles in renal fibrosis, in different renal areas of diabetic rats: glomeruli (G), proximal and distal convoluted tubules (PCT; DCT). Methods: Male Sprague Dawley rats were instilled with 55 mg/kg streptozotocin (diabetes mellitus type I model, DM group) or citrate buffer (control group). Kidney samples were collected 2 weeks and 2 months after DM induction and processed for immunohistochemistry. Results: Diabetic animals showed higher Wnt4 kidney expression both 2 weeks and 2 months post-DM induction, while Snail expression significantly increased only 2 weeks after DM initiation (*p* < 0.0001). We determined significantly higher expression of examined EMT-related genes in different kidney regions in diabetic animals compared with controls. The most substantial differences were observed in tubular epithelial cells in the period of 2 weeks after induction, with higher Snail and Wnt4 expression in PCT and increased Snail and Notch2 expression in DCT of diabetic animals (*p* < 0.0001; *p* < 0.001). Conclusion: The obtained results point to the EMT-related factors Snail, Wnt4, and Notch2 as a potential contributor to diabetic nephropathy development and progression. Changes in their expression, especially in PCT and DCT, could serve as diagnostic biomarkers for the early stages of DM and might be a promising novel therapeutic target in this condition.

## 1. Introduction

According to the latest statistical analyses, the global diabetes mellitus (DM) prevalence in 2021 was estimated at 10.5% in adults between 20–79 years of age, with a growth prediction of 12.2% by 2045 [1]. Diabetic nephropathy (DN) is a complex, slowly progressive syndrome that develops over time and consists of different histological features such as glomerular basement membrane thickening accompanied by loss of podocytes, hypertrophy of mesangial cells, and finally, renal fibrosis [2,3]. These glomerular changes with glomerular filtration function loss and subsequent microalbuminuria happen relatively late from the disease onset. They are recognized as a synonym for diabetic kidney disease (DKD), with approximately 40% of patients with DM developing it [2]. On the other hand, proximal convoluted tubule (PCT) dysfunction occurs early in DN development and may precede glomerular damage [4]. Moreover, recent studies have revealed that the distal convoluted tubular (DCT) cells might be the primary site of damage in DN [5].

The epithelial to mesenchymal transition (EMT) is a compounded, highly dynamic process in which phenotypic transdifferentiation of epithelial cells into cells with mesenchymal phenotypes occurs [6]. During the EMT, the loss of epithelial proteins, such as zonula occludens protein-1 and epithelial (E)-cadherin, coincides with the acquisition of markers that represent mesenchymal phenotype, like α-smooth muscle actin (α-SMA), vimentin and fibronectin [7]. Partial EMT of tubular epithelial cells, where they co-express both mesenchymal and epithelial markers and are capable of producing a large amount of extracellular matrix with consequent destruction of normal kidney architecture, has been recognized as one of the essential pathological mechanisms and a key contributor to the glomerular and interstitial kidney fibrosis in DKD [8]. The process of tubular EMT is mediated via a series of paracrine molecules and sophisticated intracellular signaling pathways. Wnt, Notch, and TGF-β developmental pathways are believed to be crucial in activating EMT-related transcription factors, such as Snail, Twist, Slug, and are thought to be the master regulators of myofibroblast activation and a key culprit in renal fibrosis [6]. The complete molecular and pathogenetic mechanisms underlying the role of tubular EMT in early diabetic nephropathy and DKD advancement remain mostly unidentified.

Snail is a potent transcription factor crucial for numerous biological processes but is also known as a key regulator of EMT [9,10]. Snail mediates podocytes and tubular cells EMT in the kidney and has a central function in renal interstitial fibrosis [11]. Therefore, the relationship between Snail expression and various progressive chronic nephropathies has been well established, including DN [12].

Wnt4 is a component of a large family of secreted proteins that exerts its different cellular functions through at least three specific intracellular signaling pathways, including the Wnt/β-catenin pathway, which is demonstrated to be essential as a potent mediator of the tubular EMT process by inducing its target genes, such as matrix metalloproteinase 7, fibronectin and Snail [13]. Wnt/β-catenin signaling induces podocyte and mesangial cell injury in high-glucose conditions and has a pivotal role in the occurrence and advancement of DN [14].

After binding one of its five specific ligands (Delta-like ligand 1, 3, and 4 and Jagged ligand 1 and 2) to the transmembrane receptor protein Notch (Notch 1–4), the Notch signaling pathway plays an important role in kidney development during embryogenesis. Consequently, mutations of the Notch ligands or their receptors have previously been connected with several kidney developmental abnormalities [15]. An increase in Notch signal transduction in the adult kidney induces podocytes and tubular epithelial cell apoptosis, mesangial proliferation, and glomerular sclerosis, thereby promoting the EMT process and accelerating renal interstitial fibrosis in different forms of chronic kidney disease, as well as DKD [16,17].

Since the conversion of epithelial cells to cells with mesenchymal features has been recognized as one of the crucial causes of renal fibrosis in DN, and existing treatments cannot control the progression of DKD satisfactorily, we aimed to explore the changes in the immunohistochemical expression of EMT-related factors Snail, Wnt4, and Notch2 in the renal cortex structures of diabetic rats during ageing, which could help to elucidate their impact on DN development and may serve in the design of new treatment modalities for this condition.

## 2. Materials and Methods

### 2.1. Ethics

The experimental protocol of this study was approved by the Ethics Committee of the University of Split School of Medicine, no. 16/4–09, and the study was conducted according to the guidelines of the Declaration of Helsinki.

### 2.2. Experimental Animals

In this study, experimental animals were provided by the animal facility of the University of Split School of Medicine. Male Sprague Dawley rats, weighing between 160 and 180 g, were used and held under controlled conditions (controlled temperature of 22 ± 1 °C and a light program consisting of 12 h of dark and 12 h of light).

For the induction of the diabetes mellitus type 1 (DM) model, animals received, after overnight fasting, an intraperitoneal injection of 55 mg/kg streptozotocin (STZ) freshly dissolved in citrate buffer at a specific pH value of 4.5. The control group (C) received citrate buffer in the peritoneal cavity. The rats were fed a regular laboratory diet ad libitum (4RF21 GLP, Mucedola, Settimo Milanese, Italy) comprising 64% carbohydrates, 27% proteins, and 9% fat. In order to verify diabetes in rats, plasma glucose and body mass were regularly measured. One drop of blood was collected from the tail vein, and a blood glucose level was measured by a single-touch glucometer (OneTouchVITa, LifeScan, High Wycombe, UK). Rats were considered diabetic with a glucose level above 16.5 mmol/L and included in further experiments. Glucose values were between 16.5 −30 mmol/L during the whole experiment. All rats in the experimental group, after the induction with streptozotocin, developed the symptoms of diabetes (polyuria, polydipsia, and high glucose concentration). The weight of the diabetic animals at the onset of the experiment was 160 ± 2 g, after two weeks, 208 ± 9 g, and at the end (after two months) 244 ± 7 g. The non-fasting plasma glucose concentrations were 27 ± 3 mmol/L (2 weeks) and 27 ± 2 mmol/L (2 months). The weight of the control animals at the onset of the experiment was 167 ± 15 g, after two weeks, 250 ± 6 g, and at the end (after two months), 466 ± 13 g. The non-fasting plasma glucose concentrations were 7.5 ± 0.5 mmol/L (2w) and 7,2 ± 0,4 mmol/L (2 months). Diabetic rats were assigned into groups based on the duration of diabetes from the point of injection of STZ to the end of the experiment: 2 weeks and 2 months (DM 2w and DM 2M). Each diabetic group matched the comparable control group (C 2w and C 2M). There were six animals for each required age in every diabetic and control group.

### 2.3. Tissue Collection and Immunohistochemistry

Isoflurane (Forane, Abbott Laboratories, Queenborough, UK) was applicated to anesthetize the rats. Next, animals were perfused with 300 mL of Zamboni’s fixative (4% paraformaldehyde and 1.5% picric acid in 0.1 M phosphate-buffered saline (PBS)) and washed with 300 mL of saline at a pH value of 7.4. Kidney samples were collected, post-fixed in the fixative as mentioned earlier, and afterward embedded in paraffin blocks for further examinations. The paraffin blocks were cut into 5 µm thick sections, as described previously [18]. Following deparaffinization, the sections were rehydrated using water and ethanol and then briefly rinsed with distilled water. Afterward, the sections were heated in a water steamer with sodium citrate buffer, pH value of 6.0, for 30 min. After cooling to room temperature, the sections were incubated with primary antibodies (Table 1).

Also, we have used anti-vimentin and anti-α-SMA as markers of the mesenchymal phenotype, which expression is implicated in EMT-related renal fibrosis (Table 1). Then, sections were left overnight in a humidified chamber at room temperature. After rinsing with PBS, the sections were incubated for 60 min with a combination of secondary antibodies (Table 1). 4,6-diamidine-20-phenylindole dihydrochloride (DAPI) was used for nuclear staining. The collected kidney sections were examined and photographed under a BX51 microscope (Olympus, Tokyo, Japan), and images were then processed with CellA Imaging Software for Life Sciences Microscopy (Olympus). Kidney sections were analyzed within three different areas: glomerulus (G), proximal convoluted tubule, and distal convoluted tubule focusing on the nuclei and cytoplasm of examined cells and the membrane part of captured tubules. For further analysis, 20 non-overlapping fields were captured using ×40 objective magnification. All glomeruli, PCT, and DCT on captured fields were examined. The number of Snail, Wnt4, Notch2, vimentin, and α-SMA immunoreactive cells was calculated and expressed as a percentage of total cells. The staining intensity of positive cells was semi-quantitatively classified at four degrees: the absence of any reactivity (−), mild reactivity (+), moderate reactivity (++), and strong reactivity (+++). Three investigators analyzed the images independently. Interrater agreement was tested with interclass correlation analysis, which yielded a coefficient >0.75, indicating excellent agreement [19].

Microphotographs were examined using ImageJ software (National Institutes of Health, Bethesda, MD, USA). The number of positive cells were calculated, determined by any level of color staining intensity (green for Snail, red for Notch2 and Wnt4) in the nuclei, perinuclear area, cytoplasm, and membrane in renal samples. The percentage of positive cells in the three analyzed kidney structures (glomeruli, proximal, and distal convoluted tubule) was compared between the control and experimental rat groups at each time point. After separate analyses were performed for the different kidney cortical structures, data were pooled for all areas of the control or diabetic animals and afterward re-analyzed.

### 2.4. Statistics

For statistical analysis, two-way ANOVA followed by Tukey’s multiple comparisons test and unpaired t-test were used to examine distinctions and define statistical differences between diabetic and control groups after the prior testing of data distribution. Results were expressed as a mean ± standard deviation with *p* < 0.05 for statistical significance.

## 3. Results

This study analyzed the immunohistochemical expression of EMT-related factors Snail, Wnt4, and Notch2 in the kidney cortical structures (glomeruli, proximal and distal convoluted tubules) between the control and diabetic groups during periods of two weeks and two months after DM type 1 induction.

**Figure 1:**
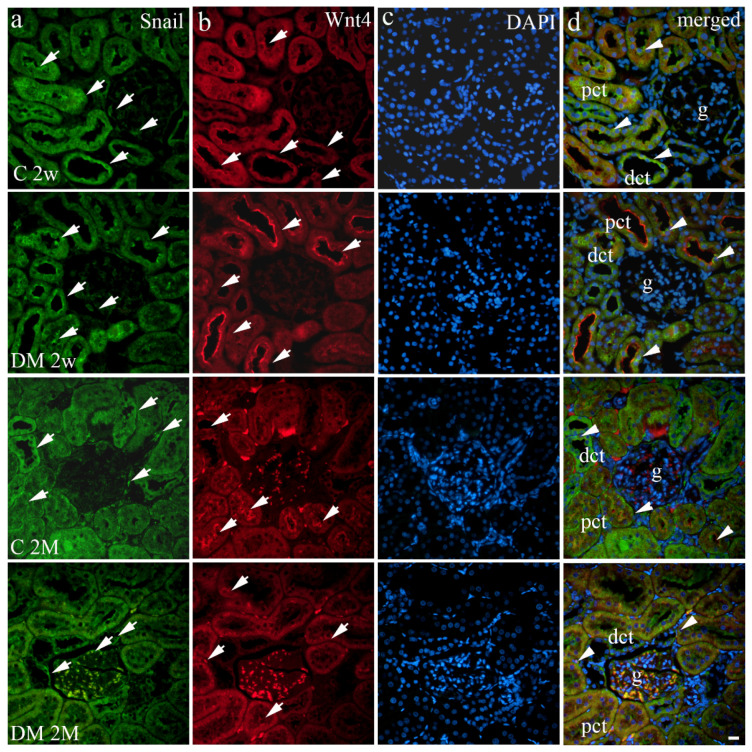
Immunohistochemical expression patterns of Snail (**a**) and Wnt4 (**b**) markers in the kidneys of diabetic (DM) and control (C) rat groups at 2 weeks (2w) and 2 months old (2M). Positive cells were observed as cytoplasmic or membrane staining (arrows) through the renal cortex. (**c**) DAPI nuclear staining. Co-expression of these markers is indicated with arrowheads (**d**). g—glomerulus, pct—proximal convoluted tubules, dct—distal convoluted tubules. The scale bar is 20 μm, which refers to all images.

Snail was mostly expressed, with mild to moderate intensity (Table 2), in the cytoplasm and perinuclear area of glomerular and tubular epithelial cells in both investigated groups (Figure 1a), whereas Snail was expressed on the membranes of examined tubules predominantly in the diabetic group, with strong reactivity.

**Figure 2:**
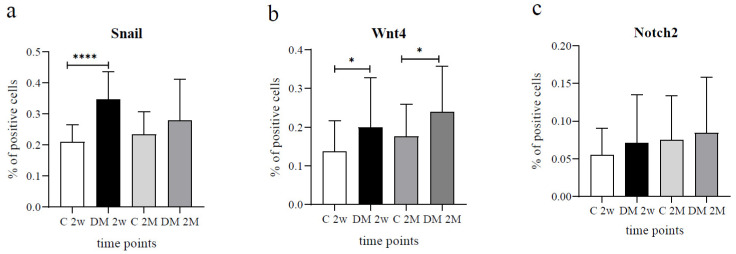
Percentage of Snail (**a**), Wnt4 (**b**), and Notch2 (**c**) positive cells in diabetic (DM) and control (C) rat groups at 2 weeks (2w) and 2 months (2M). Data presented as mean ± SD, unpaired *t*-test. * *p* < 0.05, **** *p* < 0.0001.

Diabetes induction revealed a higher Snail expression two weeks after diabetes presentation, with 34.67% positive cells compared to 20.92% positive cells in the control rat group (*p* < 0.0001). On the contrary, no significant difference was observed in Snail kidney expression between the two groups in two months (Figure 2a).

**Figure 3:**
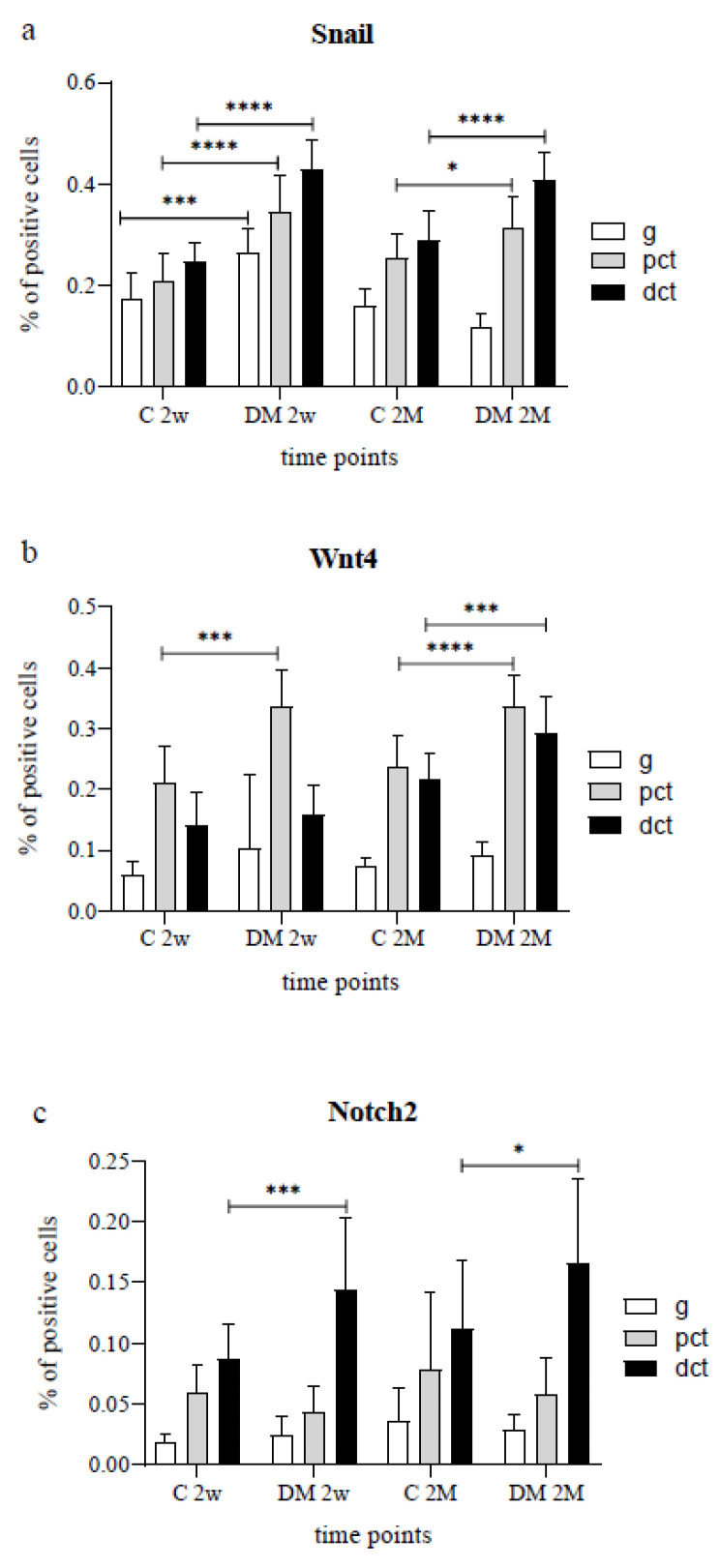
Percentage of Snail (**a**), Wnt4 (**b**), and Notch2 (**c**), positive cells in nephron substructures between diabetic (DM) and control (C) rat groups at the periods of 2 weeks (2w) and 2 months (2M). Data presented as mean ± SD, two-way ANOVA. g—glomerulus, pct—proximal convoluted tubules, dct—distal convoluted tubules. * *p* < 0.05, *** *p* < 0.001, **** *p* < 0.0001.

Regarding the total percentage of Snail positive cells, a relevant difference was seen among the kidney structures between groups too (*p* < 0.05, Figure 1a). Diabetic glomeruli, two weeks after the initiation of DM, showed significantly higher Snail expression than the glomeruli of control animals (*p* < 0.001, Figure 3a). Furthermore, Snail was even more eminently expressed in the tubular epithelial cells (PCT, DCT) of diabetic animals with 34.46% and 42.99% of positive cells, compared to the 20.81% and 24.82% of positive cells in control rat groups in the same period of two weeks (*p* < 0.0001, Figure 3a). Since the Snail expression in the cytoplasm of glomerular cells of diabetic animals decreased significantly with time and diabetes duration (*p* < 0.0001), there were no observed differences in glomerular Snail expression between control and diabetic groups after two months (Figure 3a). Consequently, two months after DM-induction, a higher proportion of Snail-positive cells was observed only in the PCT and DCT (*p* < 0.05) of the DM group (Figure 3a).

Diabetic animals showed a higher proportion of Wnt4 positive cells compared to control animals at both examined time points (19.90% vs. 13.72% at 2 weeks and 23.97% vs. 17.58% of positive cells in experimental groups at 2 months) (*p* < 0.05, Figure 2b). Wnt4 was strongly expressed predominantly on the membranes of the PCT and moderately in the DCT of control and DM groups (Table 2, Figure 1b). In contrast, glomerular Wnt4 expression remained at a very low level and mild intensity in both control and diabetic animal groups throughout the analyzed time (Figure 1b). A significant difference was observed only in the PCT two weeks after DM presentation (*p* < 0.001, Figure 3b) in favor of the diabetic group, which revealed a greater proportion of Wnt4 positive cells (33.54% vs. 21.25%). Additionally, two months after initiation, Wnt4 expression in the PCT was repeatedly higher in the diabetic group (*p* < 0.0001, Figure 3b) than in their respective controls. Moreover, the DCT of diabetic animals showed a higher percentage of Wnt4 positive cells at the same time point (*p* < 0.001, Figure 3b), whereas no significant difference was noticed in Wnt4 expression in glomeruli between the experimental groups mentioned before.

DAPI nuclear staining revealed the colocalization of Snail and Wnt4 in the PCT and DCT, while there was no colocalization of Snail and Wnt4 in glomeruli (Figure 1d).

**Figure 4:**
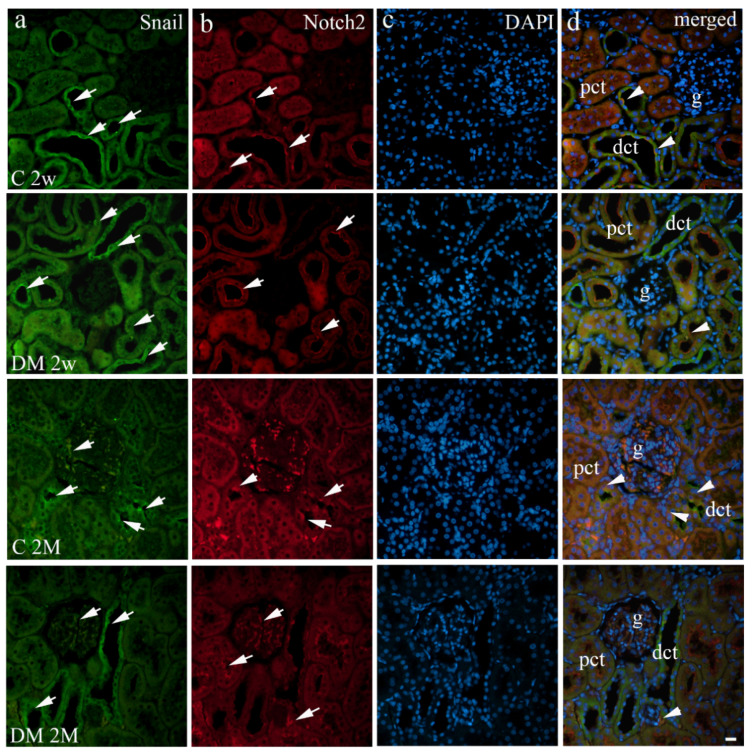
Immunohistochemical expression patterns of Snail (**a**) and Notch2 (**b**) markers in the kidneys of diabetic (DM) and control (C) rat groups at 2 weeks (2w) and 2 months old (2M). Positive cells were observed as cytoplasmic or membrane staining (arrows) through the renal cortex. DAPI nuclear staining (**c**). Co-expression of these markers is indicated with arrowheads (**d**). g—glomerulus, pct—proximal convoluted tubules, dct—distal convoluted tubules. The scale bar is 20 μm, which refers to all images.

The renal expression of Notch2 was similar between diabetic and control rat groups throughout the investigated period (Table 2, Figure 2c). Notch2 expression was observed mostly on the membranes of tubular epithelial cells in PCT and the perinuclear area of tubular cells in DCT (Figure 4b). 

Overall, both examined groups had a mainly low Notch2 immunohistochemical expression pattern in glomerular cells (Figure 3c). The glomeruli and PCT showed similar Notch2 expression between groups over time (Figure 3c). Consequently, no significant difference was detected in Notch2 expression in these renal areas between the C 2w and DM 2w groups (Figure 3c). Furthermore, there were no relevant differences in Notch2 expression in the corresponding kidney cortical structures during aging (C 2w vs. C 2M and DM 2w vs. DM 2M). A greater proportion of Notch2 positive cells was only noticed in distal tubular epithelial cells of diabetic rat groups compared to control groups at both investigated time points (*p* < 0.001), with a notably higher difference at two weeks after diabetes initiation (Figure 3c).

DAPI nuclear staining showed colocalization of Snail and Notch2 in the DCT and, to a smaller degree, in PCT. There was no colocalization of these genes in glomerular cells (Figure 4d).

**Figure 5:**
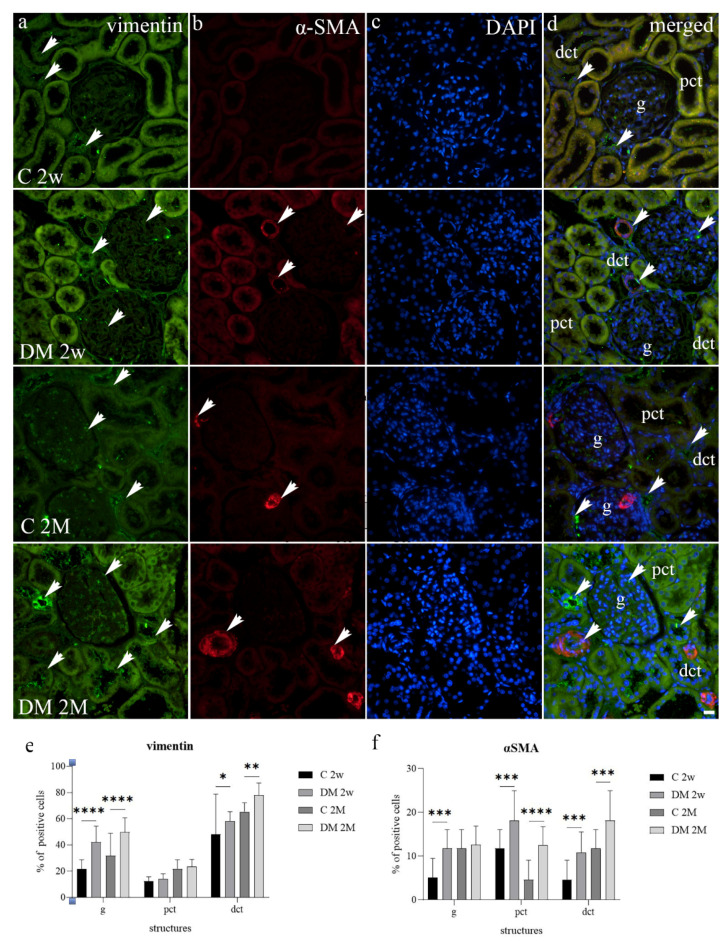
Immunohistochemical expression patterns of vimentin (**a**) and α-SMA (**b**) markers in the kidneys of diabetic (DM) and control (C) rat groups at 2 weeks (2w) and 2 months old (2M). DAPI nuclear staining (**c**). Co-expression of these markers is indicated with arrowheads (**d**). g–glomerulus, pct–proximal convoluted tubules, dct–distal convoluted tubules. Percentage of vimentin (**e**) and α-SMA (**f**) positive cells in kidney cortical structures between diabetic (DM) and control (C) rat groups at the periods of 2 weeks (2w) and 2 months (2M). Data presented as mean ± SD, two-way ANOVA. g—glomerulus, pct—proximal convoluted tubules, dct—distal convoluted tubules. * *p* < 0.05, ** *p* < 0.01, *** *p* < 0.001, **** *p* < 0.0001. The scale bar is 20 μm, which refers to all images.

Vimentin was significantly up-regulated in glomerular and distal tubular epithelial cells of the diabetic group 2 weeks and 2 months after DM induction, while proximal tubular cells showed comparable vimentin immunohistochemical expression between the two groups at both time points (Figure 5a). Increased α-SMA expression was noticed in diabetic animals at two weeks in all three analyzed nephron substructures. During the period of 2 months, the same difference was observed only in the PCT and DCT of the experimental group (Figure 5b). Regarding semi-quantitative analyses of staining intensity, vimentin was expressed with mild to moderate reactivity in the glomeruli and DCT of both examined animal groups at all observed time points (Figure 5a). In contrast, α-SMA was expressed with mild reactivity in three analyzed kidney structures in C 2w group but with moderate reactivity in DM 2w group and strong reactivity in C 2M and DM 2M group (Figure 5b).

## 4. Discussion

Stemming from previous evidence that the EMT process is critical for developing renal fibrosis and therefore promotes the progression of diabetic nephropathy to the end stages of kidney disease, changes in expression of EMT-related factors—Snail, Wnt4, and Notch2—in the kidneys of control and diabetic rats were examined in order to explore their potential role in the development and advancement of diabetic nephropathy.

Firstly, we used vimentin and α-SMA as mesenchymal markers of pathological changes in renal fibrosis in DKD. The immunohistochemical assessment of kidney samples provided results in accordance with previous reports, which have revealed elevated levels of these two markers in renal tissue of diabetic individuals [20,21]. Moreover, in our study, vimentin expression was slightly more specific to glomerular and distal tubular cell alterations, whereas increased α-SMA immunoreactivity was noticed predominantly in the proximal and distal tubular cells of diabetic animals.

Furthermore, Snail expression was located mostly in the cytoplasm and perinuclear area of glomerular and tubular epithelial cells in both investigated groups. This expression pattern correlates with the preceding findings of Snail’s relatively short cytoplasmic half-life and its nuclear translocation after it has been activated to exert its function as a member of the zinc-finger 1 transcription factor family [10,22]. Snail is a core EMT regulatory transcription factor during embryonic development and cancer metastasis but is also known to play an essential role in renal fibrosis and is associated with the development of different chronic nephropathies, including DN [9,10,11]. In the current study, two weeks after DM induction, significantly higher Snail expression was observed in all three analyzed renal cortical structures (glomeruli, proximal and distal convoluted tubules) of the whole kidney of diabetic animals compared to controls. These results could emphasize the significance of commencing changes in nephron substructures at the onset of diabetes with higher Snail expression as a potential indicator of increased intensity of the glomerular and tubular EMT process in diabetic kidneys, especially in the PCT and DCT. This increase in Snail expression in diabetic animals is in contrast to the previous study results of our laboratory, which have not shown increased Snail expression in these animals [23]. This could be partially explained due to the distinctive methodology where any level of nuclear, cytoplasmatic, and membrane signal was observed in this study. Our results agree with those obtained in the study of the influence of high glucose and hypoxia conditions on kidney Snail expression. Sumual Siska et al. have demonstrated that hypoxia and high glucose induce Snail expression in human proximal tubule cells through different signaling pathways independent of Notch signaling [24]. A higher percentage of Snail-positive cells was seen in diabetic glomeruli two weeks after DM initiation. Similar results were observed in the study of serum response factor (SRF), a DNA binding protein and transcription factor known to regulate the EMT of podocytes [25]. It has been demonstrated that the overexpression of SRF in podocyte cells significantly up-regulated Snail, which led to podocyte dysfunction. Moreover, inhibition of SRF by a specific inhibitor significantly reduced Snail expression in glomerular cells, diminishing EMT of podocytes and ameliorating proteinuria in diabetic individuals. Another study showed that, in cultured human podocytes, angiotensin II up-regulated the expression of the Snail via Notch1 activation and promoted Snail protein translocation to the nucleus, accentuating the function of angiotensin II in DKD development and annotating the considerable role of pharmacological inhibition of the renin-angiotensin system in patients with DN [22]. On the other hand, since the glomerular Snail expression in the diabetic group decreased with time and diabetes duration, glomerular cells of both investigated groups showed a similar Snail expression pattern after two months. Moreover, when we compared the control and diabetic groups two months after diabetes initiation, Snail renal expression was almost equal between groups. Throughout time, this Snail down-regulation in diabetic kidneys contrasts the persistent difference in Wnt4 expression observed between two animal groups along the investigated period. These observations advocate that, regarding Snail expression, renal tissue potentially restores from the initial hyperglycemia-induced damage and its repercussions by initiating various compensatory mechanisms, with the potential enrolment of different intracellular signaling pathways and transcription factors in glomerular EMT and renal fibrosis progression. On comparing different renal cortical structures after two months, a higher Snail expression was noticeable in the PCT and DCT of diabetic animals. Previously, glomerular basement membrane damage with subsequent microalbuminuria has been recognized as a synonym for diabetic kidney disease, while tubular cell dysfunction has not received adequate attention in this condition so far. These results accentuate the role of the Snail signaling pathway in the long-term tubular EMT process and are compatible with our previous reports that have pointed out the relevance of the proximal and distal tubular epithelial cell changes in the development of DN [5]. Additionally, Kai-Yun Fang et al. have noticed the statistically significant up-regulation of the Snail mRNA and protein expression in diabetic rat renal tubules, while a time-dependent down-regulation of Snail expression in the insulin-treated rat kidneys [26]. Moreover, numerous studies have revealed the protecting effect of different pharmacological agents and molecules, such as berberine, gluiquidone, and melatonin, on kidney function deterioration in diabetic kidney disease through inhibiting the Notch/Snail signaling pathway and attenuating renal EMT process [27,28,29].

Up to now, 19 different Wnt ligands and 15 Wnt receptors and co-receptors have been discovered, which makes it challenging to understand and define the specific function of every receptor and ligand in the complex molecular processes regulated by Wnt signaling [13]. Wnt4 is important in kidney development during embryogenesis and has a role in the cell regeneration process of tubular epithelial cells in adult kidneys [30]. Temporary activation of the Wnt/β-catenin signaling pathway stimulates tissue repair and cell regeneration after acute ischemia-reperfusion kidney injury, though its prolonged activation contributes to renal interstitial fibrosis and aggravates CKD progression [31]. The current study detected an increased Wnt4 expression in diabetic rats at two weeks and two months post DM initiation. Significantly higher Wnt4 expression was observed only in the proximal convoluted tubules in the DM group two weeks after diabetes induction. It has been known that, among the various renal cell types, proximal tubular epithelial cells are predominantly associated with oxidative stress, inflammation, and consequently the development of renal fibrosis in DKD due to the expression of sodium-glucose co-transporters, which are, through coordinating the glucose reabsorption, culpable for body glucose homeostasis maintenance [32]. This difference persisted throughout time and diabetes duration and was eminent in the PCT of the diabetic animal group after two months. In addition, as Wnt4 showed an increasing expression pattern in the DCT of diabetic animals with time, at two months, a higher percentage of Wnt4 positive cells was seen in the distal convoluted tubules of the DM group. Such a Wnt4 up-regulation implies an increased intensity of the EMT process in tubular epithelial cells in diabetic rats.

In line with our results, Weichun He et al. found an up-regulated level of different Wnt ligands, including Wnt4, in the fibrotic kidney after unilateral ureteral obstruction [33]. The dual role of Wnt/betaβ-catenin signaling in the pathogenesis of diabetic nephropathy has been described. Several studies showed an increased Wnt4 expression in renal tubular cells in diabetic rat kidneys [34,35,36]. Moreover, one recent research studies pointed out the relevance of the Wnt/β-catenin signaling increase in the occurrence and advancement of DN [13]. In their study, alkaloid trigonelline reduced high glucose-induced injury and apoptosis of mesangial cells by suppressing the activation of Wnt/β-catenin signaling in these cells with its protective effect on renal function in DKD. On the other hand, various studies reported that hyperglycemic conditions in diabetic individuals accelerated mesangial cell apoptosis by inhibiting the Wnt/beta-catenin signaling through down-regulation of Wnt4 mRNA and protein expression in these cells [37,38]. Although these findings indicate that Wnt4 is an essential modulator of renal fibrosis development, the above-mentioned inconsistent results may suggest that the maintenance of Wnt4/β-catenin signaling in a balanced state may prevent the tubular EMT process and progression of renal interstitial fibrosis in DKD, but also signify that the effect of Wnt4 could additionally be mediated through the non-canonical Wnt signaling pathway, independent of β-catenin. Similar to Notch2, glomerular Wnt4 expression was low in all groups compared to PCT and DCT, with no differences observed between the two investigated groups. Contrary to our results, others reported an increase in Wnt activity and expression in glomeruli of mouse models of different forms of CKD. In this study, Dai C. et al. stated that podocyte-specific deletion of β-catenin protected mice from adriamycin-induced podocyte injury and alleviated microalbuminuria [39]. Conversely, another research group revealed that genetic deletion of β-catenin in podocytes did not protect mice from developing DN since the decreased Wnt/β-catenin signaling was necessary for podocyte cell differentiation. However, this caused a higher rate of podocyte cell apoptosis in experimental animals [40]. Taking these contradictory results into consideration, this remains to be further investigated.

The Notch signaling pathway has an important role in cell proliferation and survival, especially during kidney embryogenesis. Consequently, mutations of the Notch ligands Jagged1 and Notch2, one of four well-known Notch transmembrane receptors, cause human kidney developmental abnormalities [15]. As expected, once kidney development is complete, the expression of Notch pathway proteins is mostly suppressed and active predominantly in the stem cell population in adult kidneys [41]. Similarly, we noticed Notch2 being found in a relatively small quantity in kidneys of both investigated groups at two analyzed time points. Moreover, the renal expression of Notch2 did not change during the investigated period, and no differences were observed between groups. These findings represent Notch2 as a non-sensitive biomarker for the renal EMT process and kidney fibrosis in DKD. On the other hand, the Notch signaling pathway is strongly increased in the podocytes, and tubular epithelial cells of patients with different forms of CKD, where it acts as a strong regulator of the master transcription factors of EMT thereby promoting the EMT process and may accelerate renal interstitial fibrosis [16,17]. Several studies have confirmed the association between the increased Notch 1, 3, and 4 expressions in renal tissue and the development of diabetic nephropathy [17,42]. Moreover, one recent study has defined Notch2 and Jagged1 as possible biomarkers for distinguishing moderate and severe stages of diabetic nephropathy [43]. Although previous studies strongly support the role of Notch signaling in the development of renal fibrosis, they did not establish the specific ligand and receptor responsible for fibrosis advancement in different pathologies, including DN. In the current study, when comparing different cortical kidney structures, the higher Notch2 expression was observed only in the DCT of diabetic animals in both investigated periods, with more prominent alterations noticed at 2 weeks after DM induction. This emphasizes the importance of the commencing damage of renal tissue occurring already at two weeks after diabetes mellitus initiation, presumably as a result of a loss of compensatory mechanisms in the affected kidney and advises the conceivable value of more intensive anti-diabetic treatment at the disease onset in order to preserve kidney function and prevent progression of DN to the end-stage CKD. Additionally, these results could imply the role of the Notch2 transmembrane receptor in the tubular EMT process of distal tubular epithelial cells in diabetic kidney disease. On the other hand, there were no observed differences between groups regarding Notch2 expression in glomeruli and PCT. These findings are consistent with the study results that showed that podocyte-specific genetic deletion of Notch1 resulted in amelioration of diabetic nephropathy, while genetic deletion of Notch2 in podocytes did not affect microalbuminuria and the progression of DKD [44].

Additional studies are required to examine changes in expression of the different EMT-related factors in the diabetic kidney, which will serve to elucidate the link between hyperglycemia and initiation and advancement of glomerular and interstitial renal fibrosis in diabetic nephropathy, but also will allow the development of new promising therapeutic agents against this global and potentially life-threatening condition.

## Figures and Tables

**Table 1:** Primary and secondary antibodies used in the experiment.

Primary Antibodies	Dilution	Secondary Antibodies	Dilution
Goat Anti-Snail (AB 53519)	1:500	Alexa Fluor AffiniPure Donkey Anti-Sheep IgG (Jackson Laboratories 713–545–003)	1:300
Rabbit Anti-Notch2 (AB 8926)	1:100	Rhodamine (TRITC) AffiniPure Donkey Anti-Rabbit IgG (Jackson Laboratories 711–025–152)	1:300
Rabbit Anti-Wnt4 Antibody (AB 91226)	1:25	Rhodamine (TRITC) AffiniPure Donkey Anti-Rabbit IgG (Jackson Laboratories 711–025–152)	1:300
Goat Anti-Vimentin (AF2105)	1:300	Alexa Fluor 488-conjugate AffiniPure Donkey Anti-Sheep IgG (H+L), (Jackson Laboratories 713–545–003)	1:300
Mouse Anti-α-SMA (M0851)	1:200	Rhodamine RedTM-X (RRX) AffiniPure Donkey Anti-Mouse IgG (715–295–151)	1:300

**Table 2:** Staining intensity of specific antibodies in the kidneys of diabetic and control rats after two weeks and two months (C 2w, DM 2w and C 2M, DM 2M); +++ strong reactivity; ++ moderate reactivity; + mild reactivity; g—glomeruli, pct—proximal convoluted tubules, dct—distal convoluted tubules.

Antibody	Snail			Wnt4			Notch2		
	g	pct	dct	g	pct	dct	g	pct	dct
C 2w	+	++	++	+	++	++	+	++	+++
DM 2w	+	+++	+++	+	+++	++	+	++	++
C 2M	+	+	++	+	++	++	+	++	+++
DM 2M	+	++	+++	+	++	+++	+	++	+++

## Data Availability

Not applicable.

## References

[1] Sun H., Saeedi P., Karuranga S., Pinkepank M., Ogurtsova K., Duncan B.B., Stein C., Basit A., Chan J.C.N., Mbanya J.C. (2022). IDF Diabetes Atlas: Global, regional and country-level diabetes prevalence estimates for 2021 and projections for 2045. Diabetes Res. Clin. Pract..

[2] Araki S. (2014). Novel biomarkers for diabetic nephropathy. Rinsho Byori. Jpn. J. Clin. Pathol..

[3] Fu J., Lee K., Chuang P.Y., Liu Z., He J.C. (2015). Glomerular endothelial cell injury and cross talk in diabetic kidney disease. Am. J. Physiol. Ren. Physiol..

[4] Russo L.M., Sandoval R.M., Campos S.B., Molitoris B.A., Comper W.D., Brown D. (2009). Impaired tubular uptake explains albuminuria in early diabetic nephropathy. J. Am. Soc. Nephrol. JASN.

[5] Dragun M., Filipovic N., Racetin A., Kostic S., Vukojevic K. (2021). Immunohistochemical Expression Pattern of Mismatch Repair Genes in the Short-term Streptozotocin-induced Diabetic Rat Kidneys. Appl. Immunohistochem. Mol. Morphol. AIMM.

[6] Hu L., Ding M., He W. (2021). Emerging Therapeutic Strategies for Attenuating Tubular EMT and Kidney Fibrosis by Targeting Wnt/beta-Catenin Signaling. Front. Pharmacol..

[7] Liang S., Yadav M., Vogel K.S., Habib S.L. (2021). A novel role of Snail in regulating tuberin/AMPK pathways to promote renal fibrosis in the new mouse model of type II diabetes. FASEB BioAdvances.

[8] Sun X.H., Xiao H.M., Zhang M., Lin Z.Y., Yang Y., Chen R., Liu P.Q., Huang K.P., Huang H.Q. (2021). USP9X deubiquitinates connexin43 to prevent high glucose-induced epithelial-to-mesenchymal transition in NRK-52E cells. Biochem. Pharmacol..

[9] Miyake Y., Nagaoka Y., Okamura K., Takeishi Y., Tamaoki S., Hatta M. (2021). SNAI2 is induced by transforming growth factor-beta1, but is not essential for epithelial-mesenchymal transition in human keratinocyte HaCaT cells. Exp. Ther. Med..

[10] Li C.F., Chen J.Y., Ho Y.H., Hsu W.H., Wu L.C., Lan H.Y., Hsu D.S., Tai S.K., Chang Y.C., Yang M.H. (2019). Snail-induced claudin-11 prompts collective migration for tumour progression. Nat. Cell Biol..

[11] Grande M.T., Sanchez-Laorden B., Lopez-Blau C., De Frutos C.A., Boutet A., Arevalo M., Rowe R.G., Weiss S.J., Lopez-Novoa J.M., Nieto M.A. (2015). Snail1-induced partial epithelial-to-mesenchymal transition drives renal fibrosis in mice and can be targeted to reverse established disease. Nat. Med..

[12] Ohnuki K., Umezono T., Abe M., Kobayashi T., Kato M., Miyauchi M., Yamamoto N., Kimura M., Toyoda M., Suzuki D. (2012). Expression of transcription factor Snai1 and tubulointerstitial fibrosis in progressive nephropathy. J. Nephrol..

[13] Mahmoud N.M., Elshazly S.M., Rezq S. (2022). Geraniol protects against cyclosporine A-induced renal injury in rats: Role of Wnt/beta-catenin and PPARgamma signaling pathways. Life Sci..

[14] Chen C., Shi Y., Ma J., Chen Z., Zhang M., Zhao Y. (2022). Trigonelline reverses high glucose-induced proliferation, fibrosis of mesangial cells via modulation of Wnt signaling pathway. Diabetol. Metab. Syndr..

[15] Penton A.L., Leonard L.D., Spinner N.B. (2012). Notch signaling in human development and disease. Semin. Cell Dev. Biol..

[16] Zhang H., Xing J., Zhao L. (2021). Lysine-specific demethylase 1 induced epithelial-mesenchymal transition and promoted renal fibrosis through Jagged-1/Notch signaling pathway. Hum. Exp. Toxicol..

[17] Jiang L., Liu X., Hu X., Gao L., Zeng H., Wang X., Huang Y., Zhu W., Wang J., Wen J. (2022). METTL3-mediated m(6)A modification of TIMP2 mRNA promotes podocyte injury in diabetic nephropathy. Mol. Ther. J. Am. Soc. Gene Ther..

[18] Racetin A., Raguz F., Durdov M.G., Kunac N., Saraga M., Sanna-Cherchi S., Soljic V., Martinovic V., Petricevic J., Kostic S. (2019). Immunohistochemical expression pattern of RIP5, FGFR1, FGFR2 and HIP2 in the normal human kidney development. Acta Histochem..

[19] Cicchetti D.V. (1994). Guidelines, Criteria and Rules of Thumb for Evaluating Normed and Standardized Assessment Instruments in Psychology. Psychol. Assess..

[20] Luo J., Jiang J., Huang H., Jiang F., Xu Z., Zhou Z., Zhu H. (2021). C-peptide ameliorates high glucose-induced podocyte dysfunction through the regulation of the Notch and TGF-β signaling pathways. Peptides.

[21] Ina K., Kitamura H., Tatsukawa S., Takayama T., Fujikura Y., Shimada T. (2002). Transformation of interstitial fibroblasts and tubulointerstitial fibrosis in diabetic nephropathy. Med. Mol. Morphol..

[22] Gagliardini E., Perico N., Rizzo P., Buelli S., Longaretti L., Perico L., Tomasoni S., Zoja C., Macconi D., Morigi M. (2013). Angiotensin II contributes to diabetic renal dysfunction in rodents and humans via Notch1/Snail pathway. Am. J. Pathol..

[23] Kostic S., Williams B., Ksouri S., Hardung L., Filipovic N., Ferhatovic Hamzic L., Puljak L., Ghahramani N., Vukojevic K. (2020). Changes in Snail and SRF expression in the kidneys of diabetic rats during ageing. Acta Histochem..

[24] Sumual S., Saad S., Tang O., Yong R., McGinn S., Chen X.M., Pollock C.A. (2010). Differential regulation of Snail by hypoxia and hyperglycemia in human proximal tubule cells. Int. J. Biochem. Cell Biol..

[25] Zhao L., Wang X., Sun L., Nie H., Liu X., Chen Z., Guan G. (2016). Critical role of serum response factor in podocyte epithelial-mesenchymal transition of diabetic nephropathy. Diabetes Vasc. Dis. Res..

[26] Fang K.Y., Lou J.L., Xiao Y., Shi M.J., Gui H.Z., Guo B., Zhang G.Z. (2008). Transforming growth factor-beta1 and Snail1 mediate tubular epithelial-mesenchymal transition in diabetic rats. Sheng Li Xue Bao Acta Physiol. Sin..

[27] Yang G., Zhao Z., Zhang X., Wu A., Huang Y., Miao Y., Yang M. (2017). Effect of berberine on the renal tubular epithelial-to-mesenchymal transition by inhibition of the Notch/snail pathway in diabetic nephropathy model KKAy mice. Drug Des. Dev. Ther..

[28] Tian H., Yang J., Xie Z., Liu J. (2018). Gliquidone Alleviates Diabetic Nephropathy by Inhibiting Notch/Snail Signaling Pathway. Cell. Physiol. Biochem. Int. J. Exp. Cell. Physiol. Biochem. Pharmacol..

[29] Liu F., Zhang S., Xu R., Gao S., Yin J. (2018). Melatonin Attenuates Endothelial-to-Mesenchymal Transition of Glomerular Endothelial Cells via Regulating miR-497/ROCK in Diabetic Nephropathy. Kidney Blood Press. Res..

[30] Boyle S.C., Kim M., Valerius M.T., McMahon A.P., Kopan R. (2011). Notch pathway activation can replace the requirement for Wnt4 and Wnt9b in mesenchymal-to-epithelial transition of nephron stem cells. Development.

[31] DiRocco D.P., Kobayashi A., Taketo M.M., McMahon A.P., Humphreys B.D. (2013). Wnt4/beta-catenin signaling in medullary kidney myofibroblasts. J. Am. Soc. Nephrol. JASN.

[32] Vallon V. (2011). The proximal tubule in the pathophysiology of the diabetic kidney. Am. J. Physiol. Regul. Integr. Comp. Physiol..

[33] He W., Dai C., Li Y., Zeng G., Monga S.P., Liu Y. (2009). Wnt/beta-catenin signaling promotes renal interstitial fibrosis. J. Am. Soc. Nephrol. JASN.

[34] Ren X., Zhu R., Liu G., Xue F., Wang Y., Xu J., Zhang W., Yu W., Li R. (2019). Effect of sitagliptin on tubulointerstitial Wnt/beta-catenin signalling in diabetic nephropathy. Nephrology.

[35] Wang W., Zhang J., Wang X., Wang H., Ren Q., Li Y. (2018). Effects of melatonin on diabetic nephropathy rats via Wnt/beta-catenin signaling pathway and TGF-beta-Smad signaling pathway. Int. J. Clin. Exp. Pathol..

[36] Xiang X., Cai H.D., Su S.L., Dai X.X., Zhu Y., Guo J.M., Yan H., Guo S., Gu W., Qian D.W. (2019). Salvia miltiorrhiza protects against diabetic nephropathy through metabolome regulation and wnt/beta-catenin and TGF-beta signaling inhibition. Pharmacol. Res..

[37] Lin C.L., Cheng H., Tung C.W., Huang W.J., Chang P.J., Yang J.T., Wang J.Y. (2008). Simvastatin reverses high glucose-induced apoptosis of mesangial cells via modulation of Wnt signaling pathway. Am. J. Nephrol..

[38] Zhu D., Yu H., He H., Ding J., Tang J., Cao D., Hao L. (2013). Spironolactone inhibits apoptosis in rat mesangial cells under hyperglycaemic conditions via the Wnt signalling pathway. Mol. Cell. Biochem..

[39] Dai C., Stolz D.B., Kiss L.P., Monga S.P., Holzman L.B., Liu Y. (2009). Wnt/beta-catenin signaling promotes podocyte dysfunction and albuminuria. J. Am. Soc. Nephrol. JASN.

[40] Kato H., Gruenwald A., Suh J.H., Miner J.H., Barisoni-Thomas L., Taketo M.M., Faul C., Millar S.E., Holzman L.B., Susztak K. (2011). Wnt/beta-catenin pathway in podocytes integrates cell adhesion, differentiation, and survival. J. Biol. Chem..

[41] Sharma S., Sirin Y., Susztak K. (2011). The story of Notch and chronic kidney disease. Curr. Opin. Nephrol. Hypertens..

[42] Ma T., Li X., Zhu Y., Yu S., Liu T., Zhang X., Chen D., Du S., Chen T., Chen S. (2022). Excessive Activation of Notch Signaling in Macrophages Promote Kidney Inflammation, Fibrosis, and Necroptosis. Front. Immunol..

[43] Al-Awaida W.J., Hameed W.S., Al Hassany H.J., Al-Dabet M.M., Al-Bawareed O., Hadi N.R. (2021). Evaluation of the Genetic Association and Expressions of Notch-2 /Jagged-1 in Patients with Type 2 Diabetes Mellitus. Med. Arch..

[44] Sweetwyne M.T., Gruenwald A., Niranjan T., Nishinakamura R., Strobl L.J., Susztak K. (2015). Notch1 and Notch2 in Podocytes Play Differential Roles During Diabetic Nephropathy Development. Diabetes.

